# Liposarcome dorsal: aspect clinique rare

**DOI:** 10.11604/pamj.2015.20.171.5880

**Published:** 2015-02-24

**Authors:** Odry Agbessi, Adil Arrob, Kamal Fiqhi, Lahcen Khalfi, Mohammed Nassih, Karim El Khatib

**Affiliations:** 1 Service de Chirurgie Plastique et Chirurgie Maxillo-faciale, Hôpital Militaire d'Instruction Mohammed V, Rabat, Maroc

**Keywords:** Liposarcome, tumeur graisseuses, tumeur maligne, adipocytes, Liposarcoma, fatty tumor, malignant tumor, adipocytes

## Abstract

Décrit la première fois par Virchow en 1860, le liposarcome est une tumeur mésenchymateuse rare. Cette rareté est relative car les liposarcomes représentent quand même 14 à 18% de l'ensemble des tumeurs malignes des parties molles et ils constituent le plus fréquent des sarcomes des parties molles. Pour la majorité des auteurs, il ne se développerait jamais sur un lipome ou une lipomatose préexistant. Nous rapportons un cas de volumineux liposarcome de la face dorsale du tronc. L'histoire de la maladie, l'aspect clinique inhabituel « de tumeur dans tumeur », l'aspect de la pièce opératoire nous fait évoquer la possibilité de la transformation maligne d'un lipome bénin préexistant.

## Introduction

Les liposarcomes sont globalement rare et leur incidence n'excède pas 2,5 cas /million d'habitants/an [1]. Cette rareté est relative car les liposarcomes représentent quand même 14 à 18% de l'ensemble des tumeurs malignes des parties molles [2]. Et ils constituent le plus fréquent des sarcomes des parties molles [3]. Ils Prédominent au niveau des membres (50%) mais peuvent se développer également au niveau du tronc (33%) [2]; nous rapportons un cas de volumineux liposarcome de la face dorsale du tronc. Ce cas est original de part l'aspect clinique inhabituel « de tumeur dans tumeur » jamais décrit auparavant.

## Patient et observation

Un homme de 43 ans tabagique non alcoolique sans autres antécédents notables consulte pour une masse indolente du dos découverte fortuitement par le patient deux ans avant la consultation. Le patient a affirmé ne pas avoir noté une augmentation de la tuméfaction depuis la découverte mais l'apparition récente d'une induration au centre de la tumeur a motivé la consultation. L'interrogatoire ne retrouve pas de facteur déclenchant en l'occurrence pas de traumatisme ni de signes associes: pas de notion d'amaigrissement récent ni de douleur.

**Figure 1:**
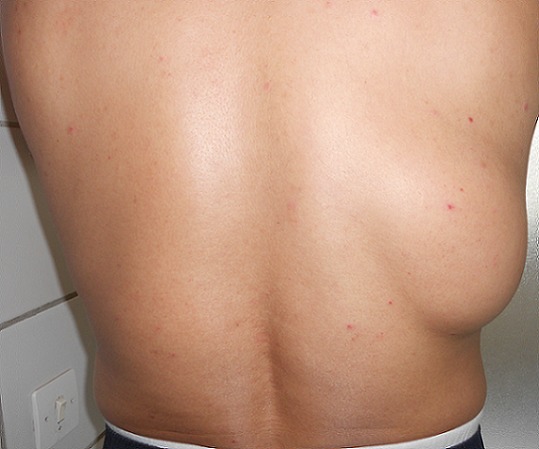
Masse tumorale du tiers moyen de la partie droite du dos (vue dorsale)

**Figure 2:**
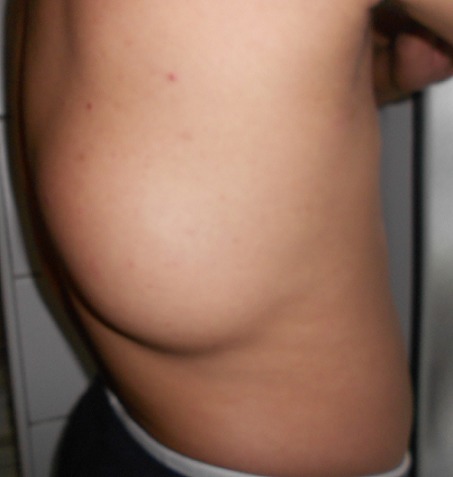
Masse tumorale du tiers moyen de la partie droite du dos (vue latérale)

**Figure 3:**
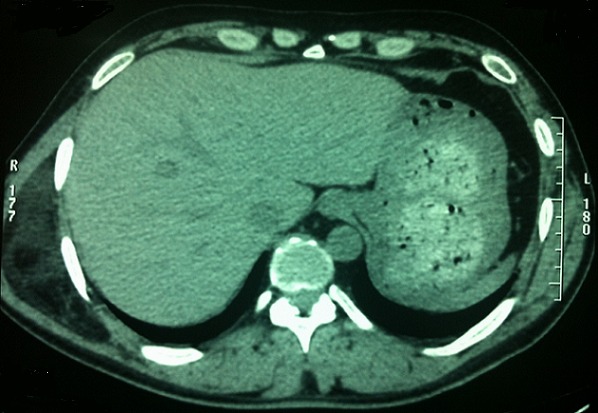
Aspect radiologique de la masse tumorale du tiers moyen de la partie droite du dos (coupe transversale)

**Figure 4:**
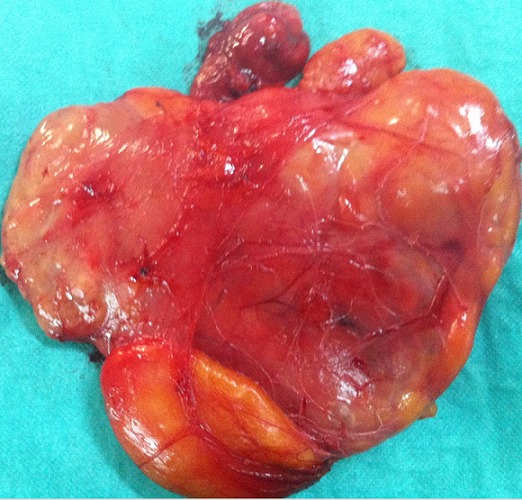
Aspect de la pièce opératoire

L'examen retrouve un patient en bon état général. Localement, une tuméfaction de la partie latérale droite du 1/3 moyen du dos. Il s'agit d'une tuméfaction ovalaire de 25 cm de grand axe (Figure 1, Figure 2). La peau en regard parait normale. A la palpation la tuméfaction est molle mal limitée et centrée par une autre tuméfaction ferme d'environ 10 cm de grand axe bien limitée mobile par rapport aux plans superficiel et profond et non douloureuse. Les aires ganglionnaires axillaires et cervicales sont libres de toutes adénopathies. Le reste de l'examen somatique était normal.. Le scanner thoracique (Figure 3) montre des images en faveur d'une masse graisseuse de la paroi thoracique droite d'allure bénigne. Le patient a bénéficié d'une exérèse tumorale sous anesthésie générale. L'incision était en s italique en regard de la masse. Après ouverture de l'aponévrose et dissection des fibres du muscle grand dorsal, la tumeur fut exposée se présentant comme un amas graisseux entourant une tumeur encapsulée elle aussi d'allure graisseuse. On fit l'exérèse de la totalité de la tumeur et de sa capsule ainsi que de l'amas graisseux périphérique (Figure 4).

L'examen anatomopathologique de la pièce opératoire montre une prolifération tumorale globalement lobulée. Les lobules sont entourés de septa fibreux d’épaisseurs variables. Au sein des plages d'adipocytes matures intra lobulaire, on note la présence de nombreux lipoblastes et des cellules adipeuses atypiques au cytoplasme multi vacuolaire avec un noyau hyperchromatique nucléolé et d'aspect encochés. Aucune nécrose tumorale n'a été notée et cette prolifération est entourée par une fine capsule fibreuse. Le diagnostic de liposarcome bien différencie de type sclérosant a été posé. Le patient a bénéficie d'un scanner thoraco abdomino-pelvienne dans le cadre d'un bilan d'extension qui a révélé des micronodules parenchymateux pulmonaires mesurant 03 mm et non spécifiques. Le patient a été présenté au staff multidisciplinaire d'oncologie ou il a été décidé de réaliser une reprise chirurgicale de la cicatrice en absence de masse résiduelle, suivie d'une radiothérapie. Apres la prise en charge du patient les suites ont été simples avec absence de récidive tumorale à ce jour (18 mois).

## Discussion

Décrit la première fois par Virchow en 1860, le liposarcome est une tumeur mésenchymateuse rare [1]. Il représente néanmoins le plus fréquent des sarcomes des parties molles [3, 4]. Il affecte le plus souvent le sujet adulte. Certains auteurs le retrouvent entre 50 et 70 ans [2, 5], d'autres délimite une période de 40 à 60 ans [3] mais on peut retenir qu'il touche l'adulte après la quatrième décade avec une prédominance masculine. Le liposarcome selon certains auteur se développe au dépends des cellules mésenchymateuses primitives plutôt qu'au dépend des adipocytes. Il ne se développerait donc jamais sur un lipome ou une lipomatose préexistant [3, 5]. Cependant il a été récemment rapporté dans la littérature un cas de liposarcome développé sur le site d'exérèse d ‘un lipome attestant que les liposarcomes peuvent naitre à partir des lipomes bénins [1]. Ceci, associé a l’évolution de la symptomatologie chez notre patient semble nous conforter dans l'idée que le liposarcome ici s'est développé sur un lipome préexistant. En effet les facteurs génétiques ou traumatiques pourraient favoriser cette différenciation des adipocytes [6]. Sur le plan clinique le liposarcome se présente sous forme d'une masse volumineuse de taille généralement supérieure à 5cm. A cet effet plusieurs auteurs rapportent un parallélisme entre la taille des tumeurs graisseuses et leur malignité avec jusqu′à 74% de sensibilité de malignité si la taille est supérieure à 5cm [7, 8]. Cette masse tumorale est d'aspect variable, le plus souvent nodulaire bien limitée [1, 3]. L'aspect clinique **« de tumeur au sein d'une autre masse tumorale »** confirmé par l'aspect per opératoire (Figure 4) observé chez notre patient n'a jamais été décrit. Ceci révèle l'originalité de notre cas et nous conforte encore une fois encore dans l'hypothèse que le liposarcome peut se développer à partir de lipome préexistant. L'altération de l’état général et les douleurs sont rares et ne se retrouvent qu'en fin d’évolution [2]. Toute tumeur graisseuse ayant donc une taille > à 5cm doit donc bénéficier d'une attention particulière et faire l'objet d'une exploration radiologique complet dans le but d'apprécier son extension tumorale et de rechercher aussi d’éventuelles métastases. A cet effet l'IRM serait l'examen le plus performant pour apprécier l'extension locale [2, 3, 9].

Sur le plan anatomopathologique on distingue cinq types de liposarcome; bien différencié, mixoide, à cellule ronde, pléomorphes, dédifférencié. Les liposarcomes de type mixoide et de type bien différencié sont les plus fréquents. [3, 6, 10]. Les liposarcomes bien différenciés sont des tumeurs de bas grade de malignité ressemblant à des lipomes d'où le nom de « lipome like » [2] avec la présence de quelques lipoblastes [3] comme le montre l'examen anatomopathologique de notre pièce opératoire. Quelque soit le type hislogique, l'exérèse chirurgicale est la clé du traitement. Elle peut, dans certains cas être complétée par une radiothérapie et parfois une chimio thérapie surtout dans les de métastases multiples ou les cas inopérables. Le pronostic du liposarcome dépend en général du type histologique, de la taille de la tumeur et de son extension au moment du diagnostic [2]. Dans tous les cas, les récidives sont fréquentes et surviennent en général dans les 24 mois qui suivent le traitement chirurgical. Les métastases sont possibles; les plus fréquentes sont pulmonaires et hépatiques et se font par voie hématogène. Notons cependant que le liposarcome de type bien différencié est de meilleur pronostic avec un taux de survie de plus de 50% à 10 ans [2, 3]. De plus une prise en charge chirurgicale initiale adéquate associée lorsque cela est indiqué à un traitement adjuvant bien conduit permet de réduire les récidives.

## Conclusion

En sommes, ce cas à travers sa présentation clinique, les aspects radiologiques et microscopique, vient renforcer l'hypothèse de certains auteurs selon la quelle un liposarcome pourrait bien se développer à partir de lipome préexistant. En outre il vient appuyer le consensus selon le quel toute tumeur graisseuse de plus de 5 cm de diamètre doit bénéficier d'une exploration radiologique complète dans le but d'affiner le diagnostic et d'optimiser la prise en charge.
